# Blunted anterior midcingulate response to reward in opioid users is normalized by prefrontal transcranial magnetic stimulation

**DOI:** 10.1038/s41398-025-03569-z

**Published:** 2025-09-03

**Authors:** Kathryn Biernacki, Rita Z. Goldstein, Malte R. Güth, Nelly Alia-Klein, Sally Cole, Suchismita Ray, Travis E. Baker

**Affiliations:** 1 Center for Molecular and Behavioral Neuroscience, Rutgers University—Newark, Newark, NJ USA; 2 National Institute on Drug Abuse, Intramural Research Program, Baltimore, MD USA; 3 Departments of Psychiatry and Neuroscience, Icahn School of Medicine at Mount Sinai, New York, NY USA; 4 Department of Biomedical Engineering, University of Minnesota, Minneapolis, MN USA; 5 Department of Psychology, Florida State University, Tallahassee, FL USA; 6 Rutgers School of Health Professions, Rutgers University—Newark, Newark, NJ USA

**Keywords:** Psychology, Neuroscience

## Abstract

Abnormalities in goal-directed behavior, mediated by mesocorticolimbic reward system, contribute to worse clinical outcomes including higher risk of treatment dropout and drug relapse in opioid users (OU). Despite efforts to counteract such neural alterations, brain-based interventions for this disorder remain ineffective. In this sham-controlled randomized study, we report the initial results on the efficacy of transcranial magnetic stimulus (TMS) in normalizing reward functioning in this population. During a reward-based choice task, we applied robot-assisted 10-Hz TMS to the prefrontal cortex in OU (Active = 16, Sham = 18) and matched healthy controls (HC, Active = 22, Sham = 24) while we recorded the reward positivity — an electrophysiological signal believed to index sensitivity of the anterior midcingulate cortex (MCC) to rewards. A robotic arm positioned a TMS coil over a prefrontal cortex target, and 50 pulses were delivered at 10-Hz before every 10 trials (2000 pulses, 400 trials). Our results revealed an interaction between TMS (Active vs Sham) and Group (OU vs HC) (F_1,72_ = 6.9, p = 0.01, η^2^ = 0.09). First, in the Sham TMS condition, OU exhibited a blunted reward positivity compared to HC (p = 0.01, d = 0.84). Second, OU receiving active TMS displayed a larger reward positivity compared to OU receiving sham (p = 0. 003, d = 0.98), and no differences were observed between OU and HC (p = 0.42, d = 0.17) or HC receiving sham (p = 0.48, d = 0.11). We envision that targeting a specific frontal-cingulate reward pathway is an important first step to maintain long-terms effect of TMS on MCC reward function, which may enhance treatment success through the maintenance of treatment goals.

## Introduction

Opioid use disorder (OUD) is widely seen as the pathological usurpation of the mesocorticolimbic reward system that normally serves several goal-directed processes (e.g., action selection and monitoring, reward valuation, decision-making) [1]. For instance, chronic opioid exposure dysregulates the ventral tegmental area (VTA), the primary source of the mesocorticolimbic dopamine neurons [1–3], leading to altered dopamine signaling, structural damage and disrupted connectivity with other reward-related brain regions [4 Notably, the midcingulate cortex (MCC), which receives its primary dopaminergic input from the VTA and plays a critical role in reinforcement learning and decision making [1, 5], exhibits both structural and functional abnormalities in people who use opioids [6–8], along with dysregulated dopamine signaling [9–12]. In parallel, a comprehensive meta-analysis indicated that opioid users (OU) demonstrate severe decision-making deficits, which have been linked to higher treatment dropout and rates of relapse, persisting years after cessation of use [13]. Together, this evidence suggests that MCC dysfunction would underlie several neurocognitive deficits and substance-related problems observed in OU, and therefore presents an opportunity for intervention. However, treatments for current or past opioid use disorder fail to address such neurocognitive deficits directly, likely due to the limited extant circuit–based treatments (e.g., with transcranial magnetic stimulation, TMS) and neurocognitive measures (e.g., with EEG, fMRI) that are yet to be fully integrated as outcome measures into clinical trials [14]. Leveraging the significant translational potential of non-invasive brain stimulation [15], we report a combined EEG and TMS study, in a randomized sham-controlled design, aimed to quantify and modulate the reward function of the MCC in OU.

It is well established that VTA dopamine neurons encode a reward prediction error (RPE) – the difference between predicted and received rewards – and it has been suggested that the MCC utilizes these RPEs to learn the value of rewards for the purpose of selecting the most appropriate action plan directed towards goals [16–18]. In this way, valuation by MCC appears to increase the motivation and effort to work for the reward (‘wanting’), as distinct from the hedonic enjoyment (‘liking’) of the reward when consumed [19]. In humans, the impact of RPE signals on the MCC can be reliably measured using the reward positivity, an event-related brain potential (ERP) that is sensitive to rewarding feedback received during decision-making tasks [20, 21]. This proposal has found support from several sources of evidence. Foremost, evidence from fMRI, combined EEG/fMRI, and intracranial recordings in rodents, non-human primates, and humans provide converging support that the reward positivity is produced in the MCC [17]. Notably, a recent study using simultaneous scalp and intracranial EEG recordings from human epilepsy patients provided direct evidence that reward positivity originates in the MCC [22]. Empirical, computational, and theoretical work also show that reward positivity amplitude aligns with the axiomatic definition of an RPE signal, such as scaling with outcome expectancy, being sensitive to valence, and exhibiting temporal specificity similar the timing of RPE signals recorded directly from the dopamine midbrain nuclei in humans [23]. Importantly, individuals with substance use disorder produce a severely blunted reward positivity to monetary rewards [19, 24, 25], a finding which dovetails with previous neuroimaging studies implicating abnormal functioning of the cingulate cortex in addiction [26–29]. Together, this evidence highlights the sensitivity of reward positivity to mesocorticolimbic reward circuit activation and underscores its role as a key biomarker of RPE signaling in the MCC and severity of reward-related deficits observed in substance use disorders.

While the MCC is too deep for TMS’ direct reach, neuroimaging studies have demonstrated that TMS can exert distant effects (on the MCC) if targeting interconnected regions [30]. Dense anatomical connections between the dorsolateral prefrontal cortex (DLPFC) and MCC [31–33] have therefore designated the DLPFC as a common TMS target, serving as an access node to the cingulate cortex. Notably, positron emission tomography studies have shown that 10-Hz TMS over the left, but not right, DLPFC significantly increases dopamine release in the cingulate cortex and striatum [34–36], an action attributed to modulating dopaminergic neuron activity in the VTA and its projection targets [34]. This proposal aligns with animal work demonstrating that stimulating glutamatergic projections from the prefrontal cortex can regulate VTA activation [15, 34, 36]. Other mechanisms may be more direct and mediated by local activation of the neuron terminals in the DLPFC and MCC [31, 37]. For example, blood flow responses in the human MCC and evoked field-potential responses in the rodent cingulate are enhanced following 10-Hz TMS of the DLPFC, an effect proposed to reflect heightened neuronal excitability and lasting synaptic efficacy in the MCC [31]. Several fMRI studies have also demonstrated that TMS to the DLPFC induces a direct increase in brain hemodynamic responses in the cingulate cortex in both healthy and substance using populations [38–40]. We recently demonstrated that applying 10-Hz repetitive TMS to the left DLPFC increased the amplitude of the reward positivity in nicotine-deprived smokers [41] and polysubstance users [25], as well as improved reward learning and decision-making in polysubstance users [25] and healthy controls [42]. These findings bolstered our decision to use 10-Hz TMS in treating reward-related MCC dysfunction in OU.

In this study, we examined whether robot-assisted 10-Hz repetitive TMS over the left DLPFC can modulate reward positivity amplitude in OU. To note, our project utilized robotic TMS for precision targeting within the TMS session to maintain a high level of experimental control when measuring dose/response relationships (see methods section). Thus, combined with the use of the reward positivity to monitor MCC reward function, robotic TMS thus provides an unprecedented opportunity to systematically study the impact of 10-Hz TMS on MCC activity in OUD during task performance, with high precision and reliability. First, we hypothesized that OU would exhibit a blunted reward positivity, relative to healthy controls. In addition, as a crucial step toward determining whether MCC abnormalities in OUD are distinct from widespread cortical atrophy, we leveraged neuroimaging data from a clinical OUD study to evaluate the structural integrity of the MCC in a well-characterized cohort of OUD patients compared to healthy controls. Second, we predicted that Active 10-Hz TMS would normalize the reward positivity in OU randomized into the Active condition, such that they would produce a reward positivity comparable to that of healthy controls. Given that previous research has not assessed the impact of TMS on reward processing in OUD, the results presented in this pre-clinical study would provide the first step toward the use of TMS in the recovery of reward-related neural dysfunction in OUD.

## Materials and methods

### Participant recruitment and assessment

Participants were recruited through the Rutgers Alcohol and Drug Assistance Program, and local advertisements placed in the Newark community. Eligible participants were scheduled to complete one experimental EEG-TMS session. On the day of testing, participants provided informed consent and were then randomly assigned to either a TMS or Sham session. All subjects met the following inclusion criteria: English speakers; males and females aged 18–55 years old; ability to provide informed written or verbal consent. Exclusion criteria for all subjects included un-correctable visual impairment, uninterruptable central nervous system medication, severe brain injury (traumatic or acquired), and TMS contraindications (e.g., pregnancy, braces, history of seizures, medication which lowers the seizure threshold). Participants were financially reimbursed for their time and all data obtained was kept strictly confidential. The study was registered online (NCT04432493, clinicaltrials.gov) and approved by the Rutgers research ethics committee and was conducted in accordance with the ethical standards prescribed in the 1964 Declaration of Helsinki.

In line with our previous work [25], problematic substance use was measured by the Alcohol, Smoking and Substance Involvement Screening Test (ASSIST) [43, 44]. To note, the ASSIST provides both a Global Continuum of Substance Risk (GCR) score^1^ and substance-specific sub-scores that identify the degree of problematic substance use for individual substances^2^. People were included in the chronic opioid-using group (OU) if they reported current or past problematic opioid use (e.g., heroin, oxycodone, hydrocodone, fentanyl) for at least 1 year, at least weekly use and/or met current criteria for opioid dependence according to the opioid-specific sub-score of the ASSIST (ASSIST score >12). People who reported past problematic opioid use had been abstinent from illicit opioid use for at least two weeks (average abstinence = 31.25 months, range = 0.5–240 months]). People in the OU group were not excluded for other drug use (see ASSIST substance use risk scores in Table 1). People were included in the healthy control group (HC) if they had no significant history of opioid use (ASSIST score <=3), or other drug-types (ASSIST score < 11 for each drug category or GCR < 16). Additionally, controls and opioid users were excluded for severe mental health issues (e.g., schizophrenia, bipolar). Given the comorbid nature of drug addiction, we collected assessments of depression (Becks Depression Inventory) and anxiety (Becks Anxiety Inventory) to test for within group differences (Active vs. Sham, and OU vs. HC) (see Table 1). The final sample consisted of 34 participants in the OU group (65% Black/African American; 29% White, 3% Asian; 3% Other or More than One Race) and 46 (39% Black/African American; 39% White, 9% Asian; 13% Other or More than One Race) participants in the HC group (see Table 1 for full participant characteristics). Subjects were randomized into either Active (OU = 16; HC = 22) or Sham TMS groups (OU = 18; HC = 24). Two subjects were excluded due to recording issues (see EEG methods below).

**Table 1:** Sample characteristics.

Demographics					
	Opioid Use Disorder		Healthy Controls		OUD vs HC
	*Active*	*Sham*	*Active*	*Sham*	
*N*	16	18	22	24	
Age (years)	42 (10)	46 (10)	37 (12)	34 (11)	*p* < 0.001
Sex (male/female/other)	11/5/0	12/6/0	10/12/0	12/11/1	
Race (*n*)					n.s.
Black/African American	10	12	7	11	n.s.
White/Caucasian	4	6	10	8	
Asian	1	0	2	2	
Other or More Than One Race	1	0	3	3	
Education (years)	12 (2)	13 (3)	15 (2)	15 (3)	*p* < 0.001
rMT^a^	72 (7)	76 (4)	72 (8)	71 (10)	n.s.
TMS side effects	1.1 (0.03)	1.0 (0.06)	1.1 (0.02)	1.1 (0.02)	n.s.
*Opioid Use Behavior*					
Opioid craving status (*n*)^b^					
Pre-TMS	3.3	3.0	1.1	1.1	*p* < 0.001
Post-TMS	3.1	2.9	1.1	1.1	*p* < 0.001
	n.s.	n.s.			
Frequency opioid use (*n*)^c^					
Weekly	1	4			
Daily	15	14			
Years opioid use	13 (10)	18 (11)			
Average abstinence (months)^d^	37.3	23.2			
Opioid-dependence risk score^d^	18 (14)	21 (13)	0 (0)	0 (0)	*p* < 0.001
*Other Drug Use Behavior*					
Other drug risk scores^e^					
Alcohol	6 (6)	9 (9)	4 (6)	5 (5)	*p* = 0.012
Cannabis	8 (9)	6 (6)	1 (3)	1 (3)	*p* < 0.001
Cocaine	4 (8)	8 (9)	0 (1)	0 (0)	*p* < 0.001
GCR	53 (26)	73 (35)	13 (19)	10 (13)	*p* < 0.001
Smoking (FTND)	2 (2)	4 (2)	1 (2)	1 (3)	*p* < 0.001
*Mental Health*					
Depression (BDI-II)^f^	14 (11)	16 (14)	8 (8)	10 (12)	*p* = 0.014
Anxiety (BAI)^g^	9 (11)	7 (8)	7 (8)	13 (12)	n.s.

^a^TMS-related side effects were evaluated by a review of symptoms (ROS) questionnaire at the end of the session.

^b^Opioid Craving Questionnaire.

^c^Includes both illicit (e.g., heroin) and licit but abused opioids (e.g., oxycodone, fentanyl, hydrocodone).

^d^Abstinence duration is reported only for those who were past users (active n = 10; sham n = 8). Note that most past users were daily users (n = 17).

^e^ASSIST substance specific scores; scores of 0–3 indicate low risk levels for substance dependence, 4–26 moderate risk, 27+ high risk; for alcohol, 0–10 indicates low risk, 11–26 moderate risk, and 27+ high risk.

^f^Beck Depression Inventory (BDI-II). Depression severity cut-offs for the BDI-II are as follows: 0–13 minimal, 14–19 mild, 20–28 moderate, and 29–63 severe.

^g^Beck Anxiety Index – Trait (BAI). Anxiety severity cut-offs for the BAI are as follows: 0–7 minimal, 8–15 mild, 16–25 moderate, and 26–63 severe.

*To note there were no significant differences between variables within the OUD group.

### Electrophysiological task: virtual T-Maze

**Fig. 1:**
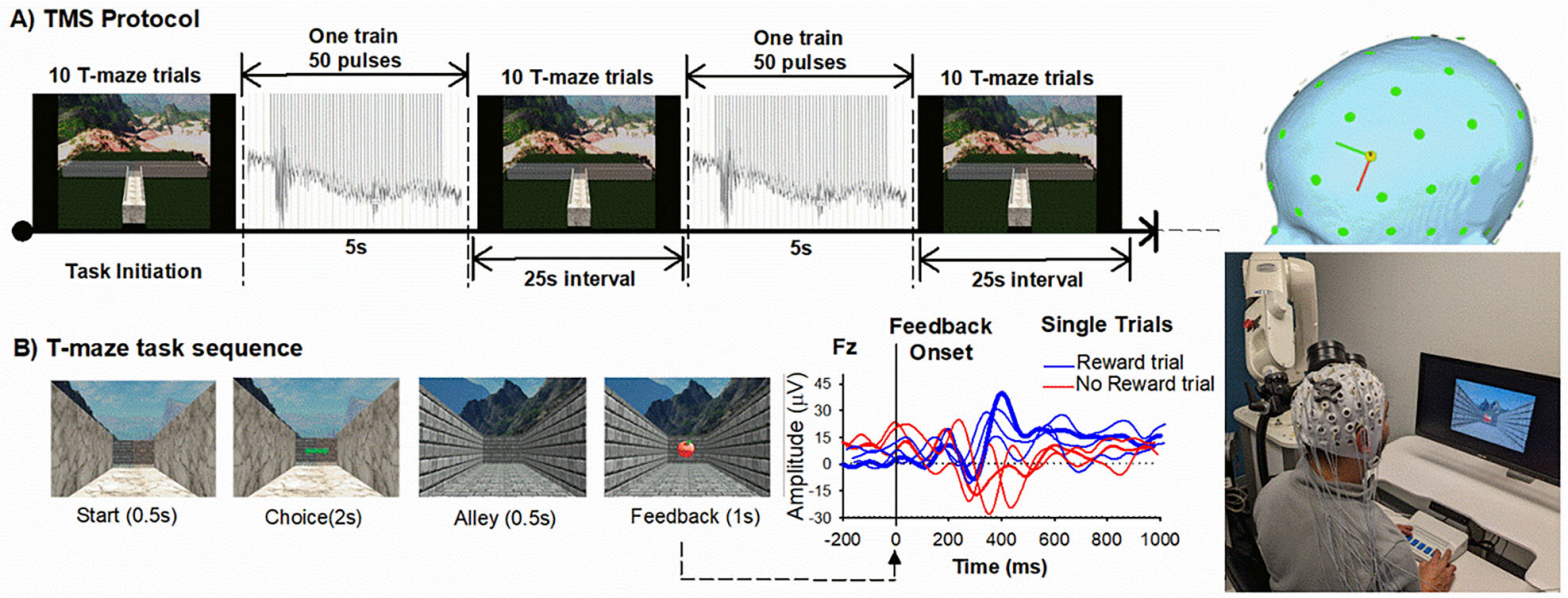
TMS protocol. **A** Displays the TMS | T-Maze Block sequence. **B** Displays a single trial sequence in the T-Maze. Right panels illustrate trial-to-trial ERPs associated with Reward (blue) and No reward (red) feedback, the robot-guided TMS set-up, and 3D head view depicting the TMS target (electrode position F3).

The reward positivity was measured using the virtual T-maze (see Fig. 1), a reward-based choice task we designed that elicits robust reward positivities [25, 45]. Participants navigated the virtual T-maze by pressing left and right buttons corresponding to images of a left and right alley presented on a computer screen. After each response, an image of the chosen alley appears, followed by a feedback stimulus (apple or orange) indicating whether the participant received 0 or 5 cents on that trial; unbeknown to the participants, the feedback is random and equiprobable. The experiment consisted of four blocks of 100 trials each separated by rest periods.

### EEG data acquisition

EEG was recorded using a montage of 23 electrodes placed according to the extended international 10–20 system [46]. Signals were acquired using Ag–AgCl ring electrodes mounted in a nylon electrode cap with a conductive gel (Falk Minow Services, Herrsching, Germany). Signals were amplified by low-noise electrode differential amplifiers with a frequency response of DC 0.017–67.5 Hz (90-dB octave roll-off) and digitized at a rate of 1000 samples per second. Digitized signals were recorded to disk using Brain Vision Recorder software (Brain Products GmbH, Munich, Germany). Electrode impedances were maintained below 10 kΩ. Two electrodes were placed on the left and right mastoids, and EEG was recorded using the average reference. For the purpose of artifact correction, the horizontal EOG was recorded from the external canthi of both eyes, and vertical EOG was recorded from above the middle of the right eyebrow and electrode channel Fp2.

### Robot-guided transcranial magnetic stimulation

With an average session duration of 30 min, accurate placement of the coil in critical for TMS protocol development and clinical efficacy [47, 48]. However, conventional TMS techniques are highly prone to human error, time intensive for both participant and investigator, and response variability is excessive within and across sessions (e.g., movement will cause the electric field pattern to change considerably) [49, 50]. Even image-based navigation with frameless stereotaxic TMS systems do not guarantee exact tilt and position, and compromise comfort, altering muscle tone over time and corrupting stimulation results [49]. To overcome these limitations, robot-assisted TMS has recently emerged as a more precise alternative to conventional TMS methods [51]. In particular, robotic TMS outperforms conventional methods by improving effect sizes, permitting investigators to test hypotheses with smaller samples, does not requiring rigid head fixation, and reduces human-to-human contact to minimize the risk of transmission of infections [49, 50, 52–55]. Near real-time head motion correction ensures consistent coil positioning, especially when involuntary movements may be problematic in clinical populations, such as in OU [49, 55]. For these reasons, our project utilized robotic TMS for precision targeting within the TMS session to maintain a high level of experimental control when measuring dose/response relationships.

A MagPro X100 with the Cool-B70 figure-of-eight coil (MagVenture, Falun, Denmark) was used as the stimulator device. Following EEG-cap set-up, resting Motor Threshold (rMT) measurements were measured via visual twitch on the contralateral (right) hand. In particular, the coil was positioned over the supposed left motor cortex area (electrode location C3) and the coil was moved in a grid like search pattern until the location at which a reproducible abductor pollicis brevis response was detected. rMT was defined as the lowest stimulation intensity, expressed as a percentage of max output of the Magstim equipment, that reliably yielded a visible muscle twitch in the hand when stimulating the hand area of the contralateral motor cortex with a single pulse (3–5 times). Stimulation intensity for each experiment was set at 110% of maximal stimulator output. Participants in the sham condition were also measured for rMT (for blinding purposes), but did not receive active stimulation for the T-Maze task. Following rMT determination, a 3D model of the subjects’ head surface was created, and the robotic arm, head model, and the tracking system was registered to a common coordinate system (SmartMove, ANT Neuro, Enschede, The Netherlands). The TMS target was then placed on the x y z coordinate corresponding to electrode location F3, and the corresponding point on the surface was selected and the orientation of the coil was calculated tangentially to cortical surface and 45° to a sagittal plane based on the head model. The virtual coil position was then transformed to robot coordinates, which defined the movement of the robot to the corresponding target position relative to the subject’s head. The position of the cranium was continuously tracked, and the trajectory of the coil’s path was adapted to head movements in real-time. This procedure guaranteed a precise <1 mm coil-to-target position and allowed free head movements during the experiment.

### Design

Using a randomized control between-subjects design, participants were randomized into either the TMS Active or Sham group (coil flipped 180° to mimic auditory stimulation) during task performance. Participants were blinded as to the condition during the experiment and received a debriefing statement at the completion of the experiment detailing which group they were randomized to. All participants completed consent protocols, and questionnaires and were then fitted with the EEG cap for ERP recording. Following the electrode cap set-up and rMT measurements, the robotic arm positioned the TMS coil over the electrode location F3, and maintained the coil position 10 mm from the scalp at a 45°angle (Fig. 1). The EEG was recorded continuously while participants freely navigated the virtual T-maze to find monetary rewards (see Fig. 1B) and participants were asked to respond in a way that maximized their rewards. The experiment consisted of four segments of 100 trials, each consisting of 10 blocks of 10 trials separated by rest periods (i.e., 4 segments × 10 blocks × 10 trials for 400 trials total; 200 reward and 200 no reward trials). From the start of the T-Maze task, participants received 50 rTMS pulses delivered at 110% at 10-Hz continuously over the predefined left DLFPC target (duration: 5 s) immediately before each block of 10 trials (duration: 30 s per block). The duration between the last pulse of the train and first trial of each block exceeded 100 msec, allowing EEG data to be collected without TMS artifacts. The total duration of the task lasted approximately 20 min (2000 TMS pulses). The Sham condition received the same protocol; however, the coil was flipped 180° to ensure that participants did not receive active simulation but received the same auditory sensation. Research staff administering TMS were not blinded to TMS condition, but all participants were blinded. No subjects reported any side effects during or after the real or sham TMS (headache, nausea, etc.).

### Data processing and analysis

EEG post-processing and data visualization were performed using Brain Vision Analyzer software (Brain Products GmbH). The digitized signals were filtered using a fourth-order digital Butterworth filter with a bandpass of 0.10–20 Hz. A 1000-msec epoch of data extending from 200 msec before to 800 msec after the onset of each feedback stimulus was extracted from the continuous data file for analysis. Ocular artifacts were corrected using the eye movement correction algorithm described by Gratton, Coles, and Donchin [56]. The EEG data was re-referenced to linked mastoids electrodes, and data was baseline-corrected by subtracting from each sample the mean voltage associated with that electrode during the 200-msec interval preceding stimulus onset. Muscular and other artifacts was removed using a ± 150-μV level threshold and a ± 35-μV step threshold as rejection criteria. ERPs were then created for each electrode and participant by averaging the single-trial EEG according to feedback type (Reward, No reward).

The reward positivity was identified following a standard difference wave approach by subtracting the average reward waveform from the average no reward waveform for every electrode and participant, resulting in an average difference wave (i.e., the reward positivity). This “difference wave” approach, which was recommended in a meta-analysis of reward positivity studies [57], is a common analysis method used in ERP research to isolate neural processes by removing sources of variance that are common to both conditions. Because positive RPEs (indicating that ongoing events are better than expected) are associated with large reward positivities, and negative RPEs (indicating that ongoing events are worse than expected) are associated with the absence of the reward positivity, the difference wave approach eliminates neural activity that is common to both positive and negative feedback stimuli under the assumption that the underlying neurocognitive processes interact linearly. In other words, the difference wave isolates the reward positivity from other ERP components and provides an indirect measure of the positive RPE signal embedded in the ERP waveform. Here, the reward positivity amplitude was determined by identifying the maximum absolute amplitude of the difference wave within a 200–400-ms window following feedback onset and evaluated at front-central electrode Fz where it reached its maximum.

A consideration of TMS modulators’ effects is also valuable in interpreting additional ERP components in the post-feedback waveform, particularly the P200 (Table S2), N200, and P300 (Table S3). The P200 was measured at Fz, FCz, and Cz using the local peak amplitude within a time window starting from 100–220 ms after feedback presentation. The N200 was quantified at Fz, FCz, and Cz using the local peak amplitude within a time window starting from 220–340 ms after feedback presentation. The P300 amplitude was measured by identifying the maximum positive-going value of the Reward and No-reward ERPs recorded at electrode site Pz, within a window extending from 300–600 ms following the presentation of the feedback stimulus.

### Exploratory cortical thickness analyses

While ERP results may indicate MCC reward dysfunction in OUD, confirming structural abnormalities is essential to interpret these findings and gain a more comprehensive understanding of brain dysfunction in OUD. We propose that cortical thickness analysis can identify morphometric patterns of cortical alterations in OUD, aiding in the differentiation between global and regional gray matter changes, and may detect subtle brain alterations that other measures, such as volume or density, might overlook. Here, we utilized an existing neuroimaging dataset to conduct a cortical thickness analysis between patients with OUD and healthy controls [58]. All participant demographic and clinical data are detailed in Ceceli et al., 2023 (Table 1). MRI scans were acquired using a Siemens 3.0 T Skyra (Siemens Healthcare) with a 32-channel head coil. T1-weighted anatomical image acquisition parameters were as follows: 3D MPRAGE sequence with 256 × 256 × 179 mm^3^ field of view, 0.8 mm isotropic resolution, repetition time/echo time/inversion time = 2400/2.07/1000 ms, 8° flip angle with binomial (1, −1) fat saturation, 240 Hz/pixel bandwidth, 7.6 ms echo spacing and in-plane acceleration (GRAPPA) factor of 2, with a total acquisition time of approximately 7 min. Following our previous methods of cortical thickness analysis [41, 59–61], all T1-weighted MRI images were processed using the CIVET pipeline (version 2.1) (www.bic.mni.mcgill.ca/ServicesSoftware/ CIVET). Briefly, native T1-weighted MRI scans were corrected for non-uniformity artifacts using the N3 algorithm. The corrected volumes were masked and registered into stereotaxic space, and then segmented into gray matter, white matter, cerebral spinal fluid and background using an advanced neural net classifier. The white matter and gray matter surfaces were extracted and resampled to a stereotaxic surface template to provide vertex based measures of cortical thickness. For each participant, cortical thickness was then measured in native space using the linked distance between the two surfaces across 81,924 vertices. Each subject’s cortical thickness map was blurred using a 20-mm full width at half maximum surface-based diffusion smoothing kernel to impose a normal distribution on the corticometric data, and to increase the signal to noise ratio.

Statistical analyses were performed using SurfStat (www.math.mcgill.ca/keith/surfstat/). Each participant’s absolute native-space cortical thickness was linearly regressed against group (OU and Healthy controls) and Time (Time 1 and Time 2: scans were separated by 3–4 months into inpatient treatment and equivalent times in HC) at each cortical point after accounting for the effects global brain volume, age, and sex. The following linear mixed model was fit to each one of the 81,924 cortical points (Y = b0 + b_1_ Group + b_2_Time + b_3_GLOBAL + Ɛ) where Y = Cortical Thickness, b0 = Y intercept, b3 = regression coefficients for effects of global thickness volume, ε = error term. For each cortical point, the coefficient of the Group and Time regressor was estimated and a resultant t-test value calculated, thereby producing a t-statistic map. A t-statistic threshold of statistical significance was established, taking into account multiple comparisons via the false discovery rate (FDR) method. The resulting thresholded maps were projected on an average surface template for visualization.

## Results

### Reward positivity

**Fig. 2:**
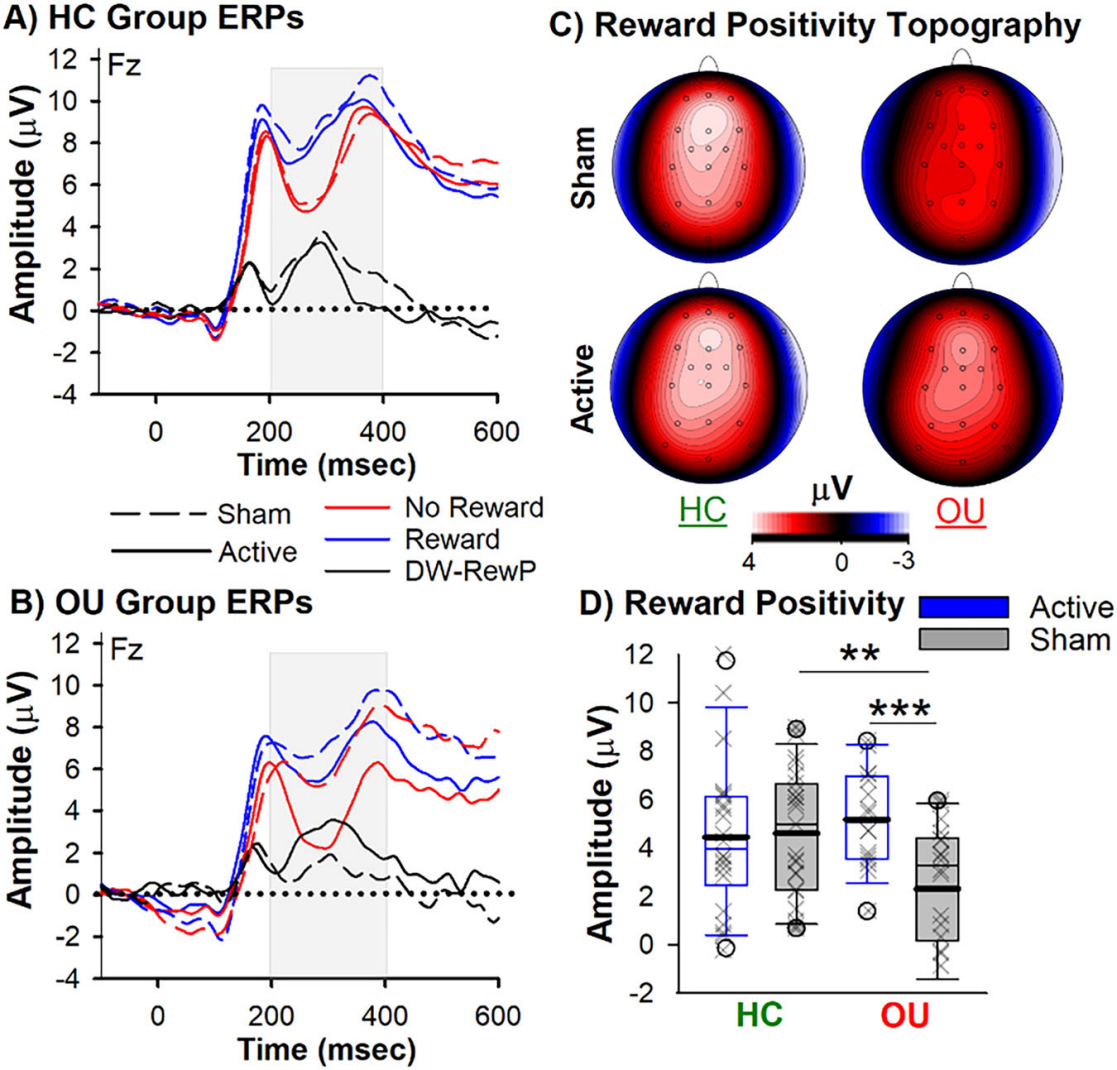
Reward Positivity Results. Grand-Averaged ERPs elicited by reward (blue) and no reward (red) feedback, and associated difference wave (black-reward positivity) for (**A**) Healthy Controls [HC] and (**B**) Opioid Users [OU] groups (Solid lines: Active TMS, Dashed lines: Sham). **C** Topoplots illustrate the reward positivity peak. **D** The box plot denotes reward positivity amplitude for the Sham (grey bars) and Active (blue bars) HC and OU groups. The boundary of the box closest to zero indicates the 25th percentile, a thin line within the box marks the median, the thick line within the box marks the mean, and the boundary of the box farthest from zero indicates the 75th percentile. Whiskers (error bars) above and below the box indicate the 90th and 10th percentiles, and symbols denotes outlying points outside the 10thand 90th percentiles. Significant effects are shown as follows: **p < 0.01, ***p < 0.005 (two-tailed).

A two-way ANOVA assessed the interaction between TMS condition (Active vs. Sham) and Group (OU vs. HC) on reward positivity amplitude (covariates included age, gender, and global drug use score [GCR]). In line with our hypothesis, this analysis revealed a main effect of TMS, F_1,72_ = 5.5, p = 0.02, η^2^ = 0.07, quantified by an interaction between TMS and Group, F_1,72_ = 6.9, p = 0.01, η^2^ = 0.09 (Fig. 2A–C). The main effect of Group did not reach significance, F_1,72_ = 1.4, p = 0.24, η^2^ = 0.01. Follow-up analyses of the TMS main effect showed that the Active TMS group displayed a larger reward positivity (mean = 4.85 μV, SEM = 0.44) compared to the Sham TMS group (mean = 3.38 μV, SEM = 0.44), p = 0.02, d = 0.54. To note, covariates (age and drug use score [GCR]) were not significant (p’s > 0.05). The covariate (Gender) was significantly associated with reward positivity amplitude, F_1,72_ = 5.7, p = 0.02, η^2^ = 0.07, indicating that female participants generally exhibited a larger reward positivity than male participants. When checking for interactions between the covariate gender and other factors in the model, the interaction between TMS and Group remained significant, F_1,72_ = 4.7, p = 0.03, η^2^ = 0.07, and no interactions with gender were significant (p’s > 0.05). This statistical check confirms that the TMS and Group interaction are robust to gender.

Post-hoc analyses of the interaction revealed two key findings. To note, post-hoc analyses were conducted using adjusted group differences after controlling for all covariates. First, within the Sham TMS group, OU exhibited a blunted reward positivity to monetary feedback (mean = 1.98 μV, SEM = 0.72) when compared to HC (mean = 4.77 μV, SEM = 0.65), t_(39)_ = 2.62, p = 0.01, d = 0.84. Second, the delivery of Active 10-Hz TMS normalized this impairment. Specifically, no differences in reward positivity amplitude were observed between the Active OU group (OU: mean = 5.13 μV, SEM = 0.72) and HC groups (Active HC: mean = 4.56 μV, SEM = 0.64), t_(36)_ = 0.82, p = 0.42, d = 0.17 | Sham HC (t_(37)_ = −0.72, p = 0.48, d = 0.11; see Fig. 2D). Importantly, the Active OU group displayed a larger reward positivity compared to the Sham OU group, t_(32)_ = −3.2, p = 0. 003, d = 0.98. No differences were observed between Active and Sham HC groups (p > 0.05: see discussion for explanation). As a check, we conducted two follow-up analysis. First, when adding depression (BDI scores) and anxiety (BAI scores) as covariates in the model, the main effect (F_1,72_ = 5.3, p = 0.03, η^2^ = 0.07) and interaction (F_1,72_ = 8.0, p = 0.006, η^2^ = 0.10) remained significant. Second, given that the OU sample consistent of varying levels of abstinence status, we conducted a separate analysis on current and past opioid users (i.e., abstinent from illicit opioid use for at least two weeks). This analysis did not change the main outcome: Active TMS group displayed a larger reward positivity (Current: mean = 5.90 μV, SEM = 0.71 | Past: mean = 4.73 μV, SEM = 0.67) compared to the Sham TMS group (Current: mean = 2.8 μV, SEM = 0.83, p < 0.05 | Past: mean = 1.66 μV, SEM = 1.2, p < 0.05). To note, there were no differences between pre- and post-TMS opioid craving scores or within and between the Active and Sham OU groups (see Table 1), and no inter-relationships were observed with our ERP measures.

### P200, N200, and P300

A consideration of TMS modulators’ effects is also valuable in interpreting additional ERP components in the post-feedback waveform, particularly the P200, N200, and P300. Here, we conducted three separate 3-way ANOVAs on P200, N200, and P300 amplitudes with Feedback (reward, no reward) as a within subject factor, and TMS and Group as between subject factors, with age, gender, and GCR as covariates. No main effects nor interaction were observed with the P200 or N200. Regarding the P300, there was a main effect of Group, (P300: F_1,72_ = 4.2, p = 0.04, η^2^ = 0.06, indicating that OU participants exhibited a smaller P300 (mean = 9.00 μV, SEM = 1.00) compared to HC (mean = 12.3 μV, SEM = 0.87). No other main effects or interactions were observed (p > 0.05). From visual inspection, it is apparent that the N200 elicited by no reward feedback was reduced in the Sham OU group; this impression was confirmed by independent sample t-tests, which indicated that the N200 to No Reward for the Sham OU group at channel FCz (mean = −3.76 μV, SEM = 0.65) was significantly smaller than every other group (Active OU: mean = −6.22 μV, SEM = 1.0, t(32) = 2.04, p = 0.05 | Sham HC: mean = −6.2 μV, SEM = 0.58: t(38) = 2.8, p = 0.008 | Active HC: mean = −6.1 μV, SEM = 0.77: t(39) = 2.2, p = 0.03). To note, a follow-up analysis indicated that within the Sham OU group, the amplitude of the N200 to reward feedback mirrored the N200 to no reward feedback (p > 0.05), confirming that reward feedback failed to induce the reward positivity in these individuals and replicating previous results in other SUD populations [19, 62].

#### Exploratory cortical thickness

**Fig. 3:**
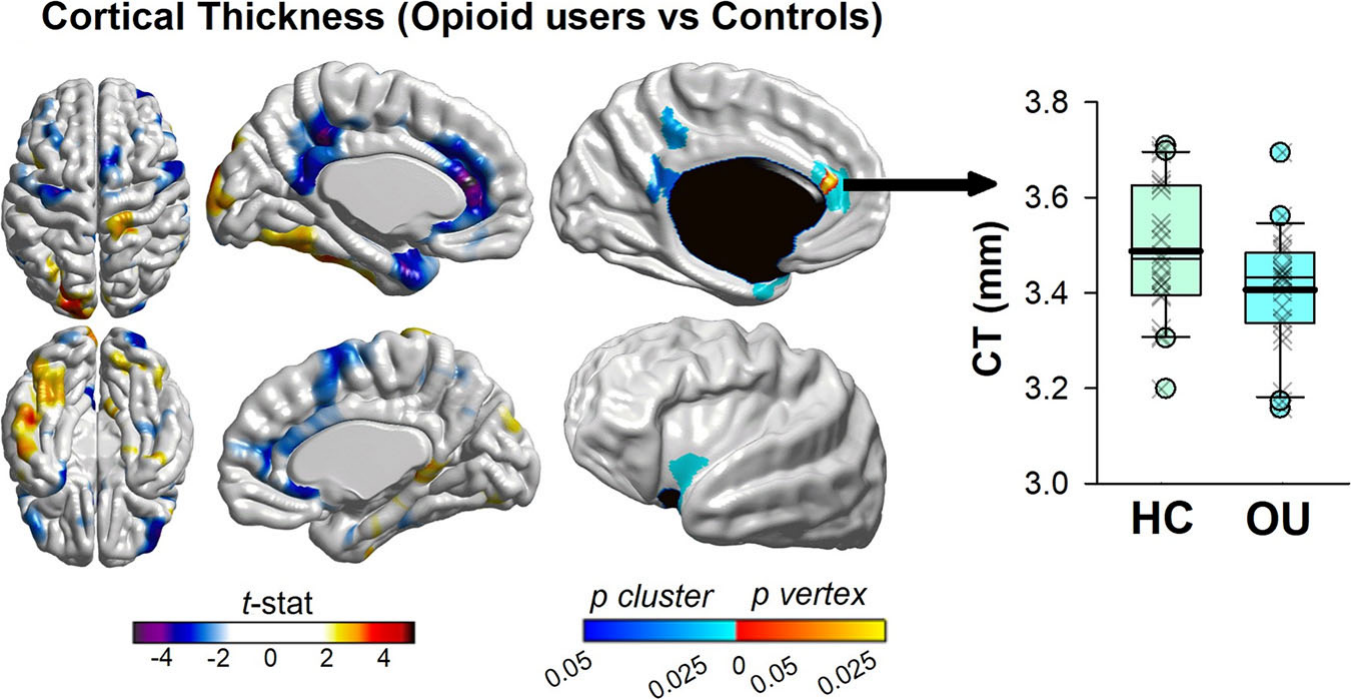
MRI-based cortical thickness estimates (re-analysis of data from [58]. Left panel: t-statistic comparing OU to healthy controls (HC) at each cortical vertex. Right Panel: thresholded map showing areas of significant differences and cluster-corrected p-value in blue (p < 0.05, FDR-corrected). Clusters of cortical thinning in OU were identified in parts of the left anterior midcingulate cortex, left insula, left precuneus region, and left retrosplenial / posterior cingulate cortex. The box plot denotes cortical thickness at the peak voxel within the MCC cluster between healthy controls and OU. The boundary of the box closest to zero indicates the 25th percentile, a thin line within the box marks the median, the thick line within the box marks the mean, and the boundary of the box farthest from zero indicates the 75th percentile. Whiskers (error bars) above and below the box indicate the 90th and 10th percentiles, and symbols denote outlying points outside the 10th and 90th percentiles.

The following linear mixed model was fit to each one of the 81,924 cortical points (Y = b0 + b_1_ Group + b_2_Time + b_3_GLOBAL + Ɛ) where Y = Cortical Thickness, b0 = Y intercept, b3 = regression coefficients for effects of global thickness volume, ε = error term (p < 0.05 FDR corrected). Over the 3–4 month period between scans, there were no significant changes in cortical thickness in either group (p > 0.05). However, regional cortical thinning was greater in OUD patients than healthy controls. Specifically, four significant clusters were identified (p < 0.05, FDR-corrected): **(1)** left ACC/MCC: nverts = 311, peak vertex xyz = −8, 37, 18 – BA32/24 **(2)** left insula: nverts = 214, peak vertex xyz = −38, 9, −12, **(3)** left posterior cingulate / precuneus region: nverts = 130, peak vertex xyz = −6, −44, 39, and **(4)** left retrosplenial / posterior cingulate cortex: nverts = 159, peak vertex xyz = −3, −46, 16. Consistent with our ERP results, these analyses revealed that the MCC exhibited the greatest cortical atrophy in OU relative to healthy controls (see Fig. 3).

## Discussion

The MCC is one of the main cortical targets of the mesocorticolimbic reward system originating in the VTA, a critical neural locus for opioid reward and addiction [1, 63]. Extensive empirical work has detailed the potentiating effects of addictive substances on the reward function of the mesocorticolimbic dopamine system. A recent proposal suggests that the enhancement of dopamine release triggered by opioid use can resemble a positive RPE: whereas the dopamine response to the reward itself disappears when the reward is predicted, transient increase in dopamine from addictive substances, like opioids, mimic positive RPEs even when drug-related events are expected [64], potentiating the value of drug-associated cues, reinforcing drug-seeking behavior, and desensitizing the system to nondrug rewards [64, 65]. Consistent with these findings and theoretical models, our current electrophysiological data show that individuals who currently misuse opioids, or misused opioids in the past, exhibited a blunted reward positivity — an electrophysiological signal that indexes sensitivity of the MCC to positive RPE signaling. These findings indicate that small monetary rewards failed to induce an MCC reward response in these individuals, replicating previous work in other substance use disorders ([41, 66]), while also supporting previous work demonstrating functional abnormalities of the MCC in opioid users [67, 68]. Further, a separate cohort of inpatients with OUD [58] demonstrated significantly greater regional reduction in cortical thickness than controls. This was observed mostly in MCC, but also in parts of the left insula, posterior cingulate cortex and precuneus — regions largely belonging to the mesocorticolimbic reward system. Together, these results suggest that abnormal indicators of MCC structure and function in OU reflect intrinsic impairments to the MCC itself.

To counteract the aberrant MCC reward function in OU, we applied 2000 pulses of 10-Hz TMS to the left DLPFC using a robot-assisted TMS system [25, 41]. Current evidence suggests that stimulating the left DLPFC using high-frequency TMS (e.g., 10-Hz) can enhance dopamine release, neuronal activity, and cerebral blow flow in the cingulate cortex, a modulatory effect mediated by local coactivation of the DLPFC and MCC [31, 69], and/or indirect modulation of the VTA, resulting in a net increase in dopamine transmission among its frontal–striatal targets [15, 34]. In line with our previous findings, applying 10-Hz TMS to the left DLPFC normalized the MCC reward response, as revealed by a quantifiable increase in reward positivity amplitude in the OU group receiving Active TMS compared to Sham TMS. Importantly, the reward positivity in the Active OU group was at par with the reward positivity in both the Active and Sham healthy control groups, indicating that the reward positivity was normalized. Furthermore, the effect of TMS was largely isolated to the predicted ERP component, the reward positivity, and was independent of abstinence status or individual differences related to age, sex, substance use and mental health status.

Together, the reward positivity findings indicate that potentiating dopaminergic RPE signaling in the MCC with 10-Hz TMS enhanced the valuation of monetary rewards in OUs, normalizing the MCC reward function. This idea dovetails previous research suggesting that DLPFC stimulation indirectly increases activity of the VTA, and subsequently its dopamine signaling to its projected targets, such as the cingulate and striatum [15, 34, 36, 70]. Indeed, our own work has demonstrated that active TMS applied to the left DLPFC enhances reward learning that is tied to dopaminergic RPE signaling in the striatum and cingulate cortex - key targets of the VTA [42]. It is also worth noting that the reward positivity amplitude is predictably altered by pharmaceutical manipulations of the dopamine system and is highly correlated with mesocorticolimbic reward activation [17, 21]. Together, these observations elucidate an important yet under investigated role of TMS in enhancing the reward valuation function of the MCC in OU and underscore the utility of the reward positivity as a highly sensitive biomarker of OUD severity and TMS treatment efficacy.

Furthermore, we also observed that people in the Sham OU group exhibited an attenuated N200 to no-reward feedback compared to all groups in the current study. The N200 is a frontocentral negative deflection in the ERP elicited by unexpected, task-relevant events, including both unexpected positive and negative feedback [20, 71]. Empirical and theoretical work suggests that the neural generator of the N200 is within the MCC [72, 73], the N200 amplitude reflects the level of effortful control applied by MCC over task performance [16] and provides an index of cognitive control deficits (e.g., inhibitory control) in substance use disorders [74, 75]. In line with this evidence, our findings of an attenuated N200 to no reward feedback in OU indicate that task-relevant events failed to elicit an MCC-dependent control process in these individuals. That the reward positivity and N200 can serve as independent measures of MCC processes of reward valuation and effortful control, respectively^3^, suggests that OU expresses impairments in both these functions, which is in line with the Impaired Response Inhibition and Salience Attribution (iRISA) model of addiction [27, 76, 77]. The iRISA model proposes that dysregulated networks involved in salience attribution (the reward network) and inhibitory control (the executive network) underlie drug seeking and taking, such that individuals with substance use disorder increase recruitment of these cognitive processes during the pursuit of drug-related goals, but a blunted response during non-drug-related goals, as we have shown here by an attenuated reward positivity and N200. Together, we argue that such abnormal MCC functioning would underlie decreased valuation (as revealed by the reward positivity) and effortful pursuit (as revealed by the N200) of a wide range of goal-directed behaviors and a marked narrowing of life goals to obtaining and using opioids [62], as proposed by the iRISA model [77, 78]. Importantly, we show that 10-Hz TMS increased the N200 amplitude in the OU group receiving Active TMS compared to Sham OU group. Although the N200 result was not anticipated, it does align with our previous study showing that 10-Hz TMS to the left DLPFC equivalently enhanced the amplitude of the N200 to reward and no reward feedback in nicotine users [43]. Because ERPs are a spatiotemporally smoothed version of the local field potential integrated over the cortex [79], our observation of an TMS-induced increase in N200 amplitude to no reward feedback confirms that direct stimulation of the DLPFC-MCC pathway enhanced MCC excitability, and possibly the control function of the MCC.

In summary, while theories of MCC function span a plethora of functions [80], we believe our findings are consistent with MCC’s role in the valuation and selection of goal-directed behavior [17, 18]. Accordingly, the MCC utilizes RPE signals (reflecting reward valuation) to assess how much control to allocate over task performance (reflecting effort expenditure), and that these factors manifest in two electrophysiological signals generated within MCC: the reward positivity and N200, respectively [16]. Put another way, the MCC function should underlie the ability to select and motivate effortful behaviors according to the value of rewards received during task execution. We propose that individuals with a history of opioid misuse produced a low reward value (blunted reward positivity) to small monetary incentives used in this task, which in turn resulted in reduced effortful control (reduced N200) during task performance. By extension, an unstable valuation and control function of MCC — along with structural loss to the MCC,as revealed by cortical thinning in MCC — would present itself as signature characteristics of several substance-related problems observed in current and past OU. For example, our whole-brain cortical thickness estimates indicated that the MCC displayed the greatest cortical atrophy and such structural abnormalities in MCC clearly point to local dysfunction. Further, several key cortical targets of the mesocorticolimbic reward system, namely the insula, precuneus, and posterior cingulate cortex, also exhibited high cortical atrophy, which aligns with network dysfunction in addiction proposed by iRISA [27, 76, 77], as well as the network-spread hypothesis implicated in neurodegenerative disorders (i.e., disease progression follows the neuronal connectome of the brain) [81]. For example, our previous cortical thickness estimates in Parkinson’s Disease indicated that regions exhibiting high cortical atrophy are part of a connectivity network centered around the substantia nigra, the neural loci of Parkinson’s Disease [60]. Because MRI/PET studies have shown that grey matter density in mesocorticolimbic regions, particularly the MCC, appears strongly correlated with dopamine receptor availability [82], we attribute the observed regional cortical thinning in OU to the dysregulation of VTA dopaminergic neurons by opioid misuse [5, 83] and potentially the networks in which the VTA and MCC serve [38, 77].

Although this research supports the initial use of TMS to modulate reward-related neural outcomes in OU, future research may address some of the study’s limitations. First, our subjects do not represent a sample of individuals with an opioid use disorder seeking treatment, and results remain to be replicated in these and other groups of OU (e.g., long-term abstaining, OUD with comorbid mental health disorders). To note, we observed normalization of the reward positivity in OU receiving Active TMS, regardless of whether they were currently using or had been abstinent from illicit opioid use for at least two weeks. Given that opioid use has been associated with abnormal reward network connectivity and decision-making impairments that persist into protracted abstinence [13, 67, 84, 85], we speculate our results would replicate to and have a meaningful clinical impact in both recently using OU populations and those who are in protracted abstinence. Second, while TMS had a clear impact on the reward positivity and N200 in OU, there were no differences between Sham and Active TMS in healthy controls. Given the plethora of known unknowns of TMS, any interpretation of these results is necessarily post hoc. Nevertheless, we believe these results are in line with the ‘Inverted U-shaped’ relationship between cognitive performance and prefrontal dopamine levels, where both too little and too much dopamine activity impairs performance [86]. On the one hand, these results likely suggests that 2000 pulses of 10-Hz TMS were not sufficient to modulate MCC reward activity in healthy control, which is not surprising given that their reward system was likely performing at an optimal level or close to ceiling. Thus, since cognitive systems are not linear, perhaps a stronger dose of 10-Hz TMS (3000–4000 pulses) may produce an observable effect on MCC activity. In support, we have previously shown that 600 pulses of prefrontal iTBS suppressed reward-related signaling in the MCC (reduction in reward positivity) and caused a decrease in effortful behaviour in healthy controls [59]. But on the other hand, we recently reported that 10-hz TMS improved reward learning and decision-making in polysubstance users [25] and healthy controls [42], the former showing an additional impact on ERPs while the latter did not. Thus, perhaps any modulatory impact of active TMS would likely be observed in a population with dysregulated reward functioning, whereas in healthy controls the impact of TMS may not move past an optimal level of functioning, and likely only be observed by dysregulating the system (e.g., overdosing or lesioning with TMS).

Third, given the stark difference in tactile sensation between sham and active TMS, future studies should consider adopting a double-blind procedure (e.g., using a placebo coil) to reduce participant and experimenter bias. In regard to clinical outcomes, previous studies have shown that one session or twenty daily sessions of 10-Hz TMS to the left DLPFC reduced cue-induced craving scores in people who use heroin [87, 88]. We failed to replicate the effect of TMS on pre- and post-craving scores, nor did we find an association between craving and our ERP data. Several factors could be at play, including diverse and small sample, sensitivity of the craving assessment, non-cue-reactivity task, and the number of TMS pulses and sessions. By contrast, perhaps the impact of TMS on craving is small and unreliable [89], and future studies should focus more on neurocognitive outcomes [14] or targeting brain regions implicated in cue-induced craving processes, such as the hyperdirect pathway [90, 91] or insula [92]. Future work may also consider how other mental health conditions (such as depression or trauma) interact with OU in the context of TMS intervention in samples powered for these types of analyses. Finally, while a good starting point, the conventional targeting approach used in the current study (scalp-landmark) frequently misses the DLPFC [30]. The DLPFC spans a large anatomical region and is highly variable across individuals [32, 93], thus simply placing the TMS coil on a DLPFC-based scalp landmark would be less than ideal for precision medicine. For example, we have previously shown that TMS responsiveness (as evaluated by the reward positivity) was associated with prefrontal cortical thickness, estimated connectivity tracts between the TMS target and MCC, as well as the striatum, and a greater left lateralization of the cingulum bundle [41]. These considerations highlight the need to optimize targeting techniques for DLPFC-MCC precision (e.g., individualized targets based on DLPFC structure, function, or connectivity with MCC) in order to increase the efficacy of TMS to normalize MCC activity long-term in substance use disorder [38].

## Conclusion

When applied in trains of repetitive pulses at around 5–10 Hz, TMS is thought to induce stable potentiation-type plasticity in the targeted circuit, potentially modifying activity in brain-wide networks [39, 94, 95]. Combined, our results suggest that, paired with conventional treatment programs and standard of care, modulating MCC putative function with TMS could potentially help recovering OU maintain continued motivation by assigning sufficient value to self-directed, goal-driven behaviors including those aligned with treatment goals. It remains to be tested whether modulating the MCC’s putative function in one session could be replicated across sessions and, importantly, extend beyond the laboratory to alleviate main addiction symptoms and improve clinical outcomes (e.g., enhance effortful pursuit of a wide range of goal-directed behaviors to improve treatment adherence, cognitive and social functioning, and employment rates). Nevertheless, by highlighting important OUD-relevant neurocognitive response to TMS, this research may represent an important step in this promising direction.

## Supplementary information

Supplementary material

## Data Availability

The data that support the findings of this study are available from the corresponding author (TEB) on request.

## Footnotes

1 According to cut-offs scores established in validation studies of the Alcohol, Smoking and Substance Involvement Screening Test (ASSIST), mid-range Global Continuum of Substance Risk (GCR) scores between 16 and 39 are an indication of hazardous or harmful substance use. GCR scores of 39.5 and higher suggests that the individual is at high risk of substance dependence (Humeniuk et al. 2008, Newcombe et al. [44]).

2 ASSIST substance-specific scores of 0-3 indicate low risk levels for substance dependence, scores 4-26 moderate risk, and scores 27+ as high risk; alcohol risk is defined at different levels, however, with scores 0-10 indicating low risk, 11-26 moderate risk, and 27+ high risk (Humeniuk et al., 2010).

3 Recent examinations of the reward positivity and the N200 have provided a nuanced account about their relationship, such that the N200 constitute a baseline control response by MCC to unexpected events and its amplitude is regulated up and down by positive and negative RPE signals conveyed to MCC. Thus, positive dopamine RPE signals conveyed to MCC about 300 msec after unexpected reward feedback drive a positive-going deflection in the ERP (the “reward positivity”) that cancels out or inhibits production of the N200. Because the variance in the amplitude of the N200 is driven more by unexpected rewarding events than by non-rewarding events, the reward positivity is typically operationalized as the size of the difference wave calculated between good (positive RPE) and bad (negative RPE) outcome ERPs (Holroyd et al., 2008; Baker and Holroyd [20]; Hajihosseini and Holroyd [71]).
