# Supplementary material for: Blunted anterior midcingulate response to reward in opioid users is normalized by prefrontal transcranial magnetic stimulation
Source: Transl Psychiatry. 2025 Sep 3;15:340. doi: 10.1038/s41398-025-03569-z (PMC12408835; doi:10.1038/s41398-025-03569-z)
Supplement: Supplementary file 1 — Supplementary material [file 41398_2025_3569_MOESM1_ESM.docx]

**Supplementary material**

**Internal Consistency Reliability Analysis** To assess the internal consistency reliability of the reward positivity (as measured by the difference wave), we conducted split-half reliability analysis using odd/even trial splitting across all participants. Single-trial ERPs were divided into two halves: odd trials (1, 3, 5, etc.) and even trials (2, 4, 6, etc.). For each half, we calculated individual reward positivity peak amplitude (Reward ERP minus No Reward trial) at electrode site Fz. Pearson correlations were computed between the two halves, and Spearman-Brown correction was applied to estimate full-length reliability using the formula: Corrected reliability = 2r / (1 + r), where r is the raw split-half correlation.

***Overall Sample Reliability***. Split-half reliability analysis revealed good internal consistency for the reward positivity measure across the full sample (N=79). Raw Pearson correlation between odd and even trials was .499 (p < .001), with a Spearman-Brown corrected reliability estimate of .67. This value exceeds typical RewP reliability estimates reported in the literature (.28-.37), suggesting in our virtual T-maze paradigm, which may be attributable to the robust measurement quality and engagement of the virtual T-maze task design, which requires active decision-making and may maintain participant engagement throughout the session compared to more passive paradigms.

***Subgroup Analysis*** Reliability patterns differed across experimental groups (Table S1). Healthy control participants showed good reliability in both the sham TMS group (.69) and active TMS group (.88). In contrast, OUD participants in the sham TMS group demonstrated virtually no reliability (.02), while OUD participants in the active TMS group showed improved reliability (.65). Despite the poor reliability in the OUD sham group, we were still able to detect significant TMS effects in our main analyses (TMS × Group interaction: F₁,₇₂ = 6.9, p = .01, η² = .09), which strengthens confidence in our findings since measurement unreliability typically attenuates effect sizes. Nevertheless, the small subgroup sample sizes (n=16-23) limit precision of reliability estimates, and these finding should be replicated with larger samples.

Table S1. Split-Half Reliability by Group (Fz Electrode)

| **Group** | **Raw Correlation (r)** | **p-value** | **Spearman-Brown Reliability** |  |
| --- | --- | --- | --- | --- |
| HC Sham | .523 | p<.01 | .69 |  |
| HC Active | .784 | p<.001 | .88 |  |
| OUD Sham | .009 | p =.97 | .02 |  |
| OUD Active | .486 | p<.05 | .65 |  |

Note: HC = Healthy Controls; OUD = Opioid Use Disorder.

**Discussion.** These reliability findings suggest that TMS enhances the precision of MCC reward signaling in OUD participants. Healthy controls showed consistently good reliability across both groups (.69-.88), with the active TMS group showing somewhat higher reliability. In contrast, OUD participants demonstrated a dramatic improvement from virtually no reliability in the sham TMS group (.02) to moderate reliability in the active TMS group (.65), suggesting that TMS specifically benefits disrupted reward systems and consistent with evidence that therapeutic effects operate through improved neural efficiency (Lin & Baker, 2022). From a clinical perspective, the restoration of neural consistency alongside amplitude normalization may represent a fundamental mechanism by which TMS treats OUD-related reward dysfunction.

**P200 and P300 ANOVA results**

Table S2. ANOVA Results for P200 Component *Electrode site: FCz; Time window: 100-220ms*

| **Effect** | **F** | **df** | **p** | **η²** |
| --- | --- | --- | --- | --- |
| Group (OUD vs HC) | 1.362 | 1,72 | .247 | .019 |
| TMS (Active vs Sham) | 2.560 | 1,72 | .114 | .034 |
| Feedback (Reward vs No-reward) | 0.873 | 1,72 | .353 | .012 |
| Group × TMS | 0.070 | 1,72 | .791 | .001 |
| Group × Feedback | 2.471 | 1,72 | .120 | .033 |
| TMS × Feedback | 0.243 | 1,72 | .623 | .003 |
| Group × TMS × Feedback | 2.180 | 1,72 | .144 | .029 |

*Note: All p-values are exact. η² = partial eta squared effect size. Analysis controlled for age, gender, and GCR as covariates.*

Table S3. ANOVA Results for P300 Component *Electrode site: Pz; Time window: 300-600ms*

| **Effect** | **F** | **df** | **p** | **η²** |
| --- | --- | --- | --- | --- |
| Group (OUD vs HC) | 4.249 | 1,72 | .043 | .056 |
| TMS (Active vs Sham) | 0.555 | 1,72 | .459 | .008 |
| Feedback (Reward vs No-reward) | 1.756 | 1,72 | .189 | .024 |
| Group × TMS | 0.121 | 1,72 | .729 | .002 |
| Group × Feedback | 0.463 | 1,72 | .498 | .006 |
| TMS × Feedback | 0.795 | 1,72 | .376 | .011 |
| Group × TMS × Feedback | 0.418 | 1,72 | .520 | .006 |

*Note: All p-values are exact. η² = partial eta squared effect size. Analysis controlled for age, gender, and GCR as covariates.*
